# 斑蝥酸钠维生素B6注射液临床应用专家共识

**DOI:** 10.3779/j.issn.1009-3419.2025.102.37

**Published:** 2025-10-20

**Authors:** 中国老年保健协会肺癌专业委员会

**Keywords:** 斑蝥酸钠维生素B6注射液, 专家共识, 恶性肿瘤, 临床应用, Sodium cantharidinate and vitamin B6 injection, Expert consensus, Malignant tumors, Clinical application

## Abstract

我国癌症防治形势严峻，尽管靶向治疗和免疫治疗取得了显著进展，化疗仍在综合治疗中占据基石地位，而其毒性反应是临床实践中不容忽视的挑战。斑蝥酸钠维生素B6注射液作为一种兼具抗肿瘤与减毒作用的复方制剂，在我国临床应用已逾二十年，但临床实践中对其应用尚缺乏统一规范。为此，中国老年保健协会肺癌专业委员会组织专家基于现有循证证据与临床经验制定了本共识。共识系统阐述了该制剂的药理机制、药代动力学特点，并针对其在肺癌、肝癌等多种恶性肿瘤中的临床应用提出了具体的推荐意见。本共识旨在为临床医生提供斑蝥酸钠维生素B6注射液的规范化用药指导，以促进其合理应用，最终改善肿瘤患者的疗效与生活质量。

## Abstract (en)

Cancer prevention and control in China face severe challenges. Although targeted therapy and immunotherapy have made remarkable progress, chemotherapy remains a cornerstone of comprehensive cancer treatment, and its toxic side effects pose a significant challenge in clinical practice. As a compound formulation exhibiting both antitumor and toxicity-reducing effects, Sodium cantharidinate and vitamin B6 injection has been utilized in clinical practice in China for over two decades. However, standardized guidelines for its application remain lacking in routine clinical use. To address this gap, the Lung Cancer Specialty Committee of Chinese Elderly Health Care Association organized a panel of experts to develop this consensus document, drawing on current evidence-based data and accumulated clinical experience. This consensus provides a systematic elaboration of the preparation's pharmacological mechanisms and pharmacokinetic profiles, and offers specific recommendations for its clinical application across various malignancies, including lung cancer and hepatocellular carcinoma. The primary objective of this consensus is to establish standardized guidance for clinicians regarding the appropriate use of Sodium cantharidinate and vitamin B6 injection, thereby promoting its rational application and ultimately enhancing treatment efficacy and quality of life for cancer patients.

我国癌症防控面临严峻挑战，2022年新发恶性肿瘤病例数高达482.47万例，占全球总数的近1/4，癌症防治工作任重道远^[1]^。科学家不断探索，尝试采用各种手段和方法来攻克癌症。近二十年来，新的抗肿瘤治疗药物不断突破，但化疗作为经典的系统性治疗手段，仍发挥着不可替代的基石作用。

斑蝥酸钠维生素B6注射液是斑蝥酸钠与维生素B6组成的复方制剂。其中，斑蝥酸钠兼具抗肿瘤及改善放化疗毒副反应的作用，可增强患者免疫功能，提高生活质量；维生素B6则在预防和控制化疗所致轻、中度恶心呕吐等方面具有确切疗效且安全性良好。该制剂在我国临床应用已逾二十年，积累了一定规模的中国患者应用数据，然而，临床医生对现有研究数据的解读存在差异，导致在适用人群、治疗时机、用法用量及联合方案等方面实践不一，尚缺乏统一的临床应用指导。

为此，中国老年保健协会肺癌专业委员会组织相关领域专家，基于现有循证证据及临床实践经验，通过召开多次专家会议就斑蝥酸钠维生素B6注射液临床应用的关键问题进行了深入讨论，最终形成《斑蝥酸钠维生素B6注射液临床应用专家共识》，以期为临床肿瘤医生提供科学规范的用药指导，推动临床合理用药实践，从而助力提升患者临床疗效和生活质量。

## 1 共识编制方法及过程

### 1.1 检索策略

共识检索策略以“斑蝥酸钠维生素B6注射液”作为中英文检索词，检索中国知网、万方数据、中华医学期刊网、EMBASE、PubMed、The Cochrane Library等中英文数据库中的临床及基础研究。检索时间从建库截至2025年5月31日。

### 1.2 推荐意见的形成

本共识的推荐意见经由系统化的方法形成。首先，基于对循证证据的汇总与分析，采用GRADE体系对证据质量进行分级（A：高质量；B：中等质量；C：低质量）^[2]^。在此基础上，初步拟定了关于斑蝥酸钠维生素B6注射液临床应用的8条推荐意见，并明确了其推荐强度（I级推荐和II级推荐）。随后，通过专家问卷调查与共识会议对推荐内容进行多轮研讨论证，将共识度≥80%的条目纳入共识草案^[3]^。最终版本经过专家组反复评议与修订后定稿。

## 2 药理机制及药代动力学特点

### 2.1 药理机制

斑蝥酸钠维生素B6注射液是一种由斑蝥酸钠与维生素B6通过螯合作用形成的复方抗肿瘤制剂。通过斑蝥酸钠介导的多靶点抗肿瘤机制（包括诱导DNA损伤、阻滞细胞周期、凋亡通路激活等），并结合维生素B6的代谢调节以拮抗相关不良反应，共同构成了其治疗作用的基础^[4,5]^。

斑蝥酸钠在抑制肿瘤生长、减轻放化疗毒性反应方面具有明确疗效。其作用机制已得到系统阐述，主要包括：（1）抑制肿瘤细胞DNA与RNA合成，阻断核酸的代谢过程，同时限制氨基酸吸收，从而抑制肿瘤关键蛋白合成，诱导细胞凋亡，实现对肿瘤细胞的直接杀伤与增殖抑制；（2）激活巨噬细胞、淋巴细胞及多形核白细胞，促进白细胞介素（interleukin, IL）分泌，调控T淋巴细胞亚群比例，增强机体抗肿瘤免疫应答及对肿瘤细胞的清除能力；（3）诱导骨髓造血干细胞向粒-单核细胞系分化，加速白细胞成熟，进而有效提升外周血白细胞数量^[4,6]^；（4）具有一定的镇痛作用，可缓解肿瘤患者的癌性疼痛症状。近年来研究^[7,8]^进一步揭示，斑蝥酸钠能够刺激淋巴细胞产生IL-2，增强机体免疫功能；通过抑制IL-8，影响肿瘤新生血管生成，从而阻断肿瘤转移途径。维生素B6作为水溶性维生素，在氨基酸代谢中发挥重要作用，通过促进氨基酸脱羧转化为γ-氨基丁酸，抑制中枢神经系统兴奋，从而减轻化疗引起的恶心、呕吐反应。同时，维生素B6在维持白细胞正常水平、促进造血功能、改善贫血状态以及减轻化疗相关并发症方面亦具有积极效果。

综上，斑蝥酸钠维生素B6注射液通过多靶点、多途径的协同作用，在肿瘤化疗的辅助治疗中展现出重要临床应用价值。

### 2.2 药代动力学

目前关于斑蝥酸钠维生素B6注射液的药代动力学研究数据有限。

## 3 斑蝥酸钠维生素B6注射液的临床应用

### 3.1 斑蝥酸钠维生素B6注射液的用法用量

推荐意见1：联合方案。采用斑蝥酸钠维生素B6注射液30-50 mL，加0.9%氯化钠注射液或5%葡萄糖注射液250 mL，静脉滴注，每日1次（IA）。

推荐意见2：单药方案。采用斑蝥酸钠维生素B6注射液40 mL，加0.9%氯化钠注射液或5%葡萄糖注射液250 mL，静脉滴注，每日1次（IB）。

推荐意见3：灌注方案。胸腔注入斑蝥酸钠维生素B6注射液50 mL联合其他胸腔注入药物。灌注治疗期间常规治疗（IB）。

斑蝥酸钠维生素B6注射液为复方制剂，规格为10 mL，每次10-50 mL，以0.9%氯化钠注射液或5%葡萄糖注射液250-500 mL，适量稀释后静脉滴注，每日1次。斑蝥酸钠维生素B6注射液在不同治疗方案中的用法与用量，参见表1^[7,9-46]^。

**表1:** 斑蝥酸钠维生素B6注射液在不同治疗方案中的用法与用量汇总表

联合治疗方案	治疗方案	SC/VB6注射液用法	疗程
非小细胞肺癌			
联合化疗	SC/VB6+GP/GC方案	SC/VB6 30-50 mL+0.9%氯化钠注射液或5%葡萄糖注射液250 mL，静脉滴注，*qd*，14 d；吉西他滨+顺铂/卡铂^[7,9-12]^	21 d为1个周期，共2-6个周期以上
	SC/VB6+TP方案	SC/VB6 50 mL+0.9%氯化钠注射液或5%葡萄糖注射液250 mL，静脉滴注，*qd*， 连用5 d、14 d；紫杉醇+顺铂^[13,14]^	14-21 d为1个周期，共2个周期以上
	SC/VB6+NP方案	SC/VB6 30-50 mL+0.9%氯化钠注射液或5%葡萄糖注射液250 mL，静脉滴注，*qd*，连用10 d、15 d；长春瑞滨+顺铂^[15-17]^	14-28 d为1个周期，共2-4个周期以上
	SC/VB6+DP/DC方案	SC/VB6 20-50 mL+0.9%氯化钠注射液或5%葡萄糖注射液250 mL，静脉滴注，*qd*，连用10 d、15 d；多西他赛+顺铂/卡铂^[18,19]^	21 d为1个周期，共2个周期以上
	SC/VB6+AP方案	SC/VB6 10-50 mL+0.9%氯化钠注射液或5%葡萄糖注射液250 mL，静脉滴注，*qd*，14 d；培美曲塞+顺铂^[20-22]^	21 d为1个周期，共3-4个周期
联合抗血管生成治疗	SC/VB6+重组人血管内皮抑制素注射液+GP化疗	SC/VB6 40 mL+0.9%氯化钠注射液250 mL，静脉滴注，*qd*，14 d +重组人血管内皮抑制素注射液（恩度）+吉西他滨+顺铂^[23]^	21 d为1个周期，2个周期以上
联合放疗	SC/VB6+放疗	SC/VB6 40 mL+0.9%氯化钠注射液250 mL，*qd*，5 d+放疗2 Gy/次，5 d^[24]^	5 d为1个周期，与放疗同步，持续至放疗结束
胸腔灌注	SC/VB6+顺铂	SC/VB6 40-50 mL+顺铂40 mg^[25,26]^	每周1次，治疗2-4周
	SC/VB6+洛铂	SC/VB6 50 mL+0.9%氯化钠注射液120 mL+洛铂30 mg^[27]^	每周1次，共2-4次
原发性肝细胞癌			
TACE联合治疗	SC/VB6+TACE，TACE相关化疗药物中加入SC/VB6	SC/VB6 0.5 mg/d，静脉滴注，*qd*，14 d，d3行TACE；TACE相关化疗药物中 加入SC/VB6 0.5 mg^[28]^	14 d、4-6周为1个周期，共2-3个周期
	TACE相关化疗药物中加入 SC/VB6	TACE相关化疗药物中加入SC/VB6 80 mL^[29]^	30 d为1个周期，共3个周期
	SC/VB6+TACE	SC/VB6 50 mL+5%葡萄糖注射液250 mL，静脉滴注，*qd*，14 d； d3行TACE^[30]^	4-6周为1个周期，治疗周期1-9次
TACE术后辅助	TACE术后当日	SC/VB6 50 mL+0.9%氯化钠注射液500 mL，静脉滴注，*qd*，28 d； d1静脉滴注前行TACE^[31]^	28 d
	TACE术后次日	SC/VB6 30 mL+5%葡萄糖注射液250 mL，静脉滴注，*qd*，21 d^[32]^	21 d
	TACE术后SC/VB6+索拉非尼	TACE；SC/VB6 30 mL+5%葡萄糖注射液250 mL，静脉滴注，*qd*； 索拉非尼，400 mg/次，bid^[33]^	28 d
联合放化疗	SC/VB6+低剂量放疗序贯FOLFOX4	SC/VB6 40 mL，静脉滴注，*qd*，14 d+放疗+序贯化疗 （奥沙利铂+5-氟尿嘧啶+亚叶酸钙）^[34]^	14 d为1个周期，共12个周期
联合化疗	SC/VB6+奥沙利铂+5-氟尿嘧啶	SC/VB6 40mL+5%葡萄糖注射液250 mL，静脉滴注，*qd*，14 d； 奥沙利铂+5-氟尿嘧啶注射液^[35]^	14 d为1个周期，共4个周期
联合射波刀	SC/VB6+射波刀	SC/VB6 30-50 mL+0.9%氯化钠注射液或5%葡萄糖注射液250 mL， 静脉滴注，*qd*，21 d+射波刀^[36]^	14 d为1个周期，每月1次，共2个周期
联合微波消融术	SC/VB6+MVA	MWA；SC/VB6 30 mL+0.9%氯化钠注射液250 mL，静脉滴注，*qd*，28 d^[37]^	28 d
单药治疗	SC/VB6	SC/VB6 40 mL+5%葡萄糖注射液250 mL，静脉滴注，*qd*^ [38]^	至病情进展
消化道肿瘤			
联合化疗	SC/VB6+XELOX方案	SC/VB6 40-50 mL+0.9%氯化钠注射液250 mL，静脉滴注，*qd* +奥沙利铂+卡培他滨^[39]^	14-21 d为1个周期，2个周期以上
	SC/VB6+FOLFOX4方案	SC/VB6 40 mL+0.9%氯化钠注射液500 mL，静脉滴注，*qd*，14 d +奥沙利铂+亚叶酸钙+5-氟尿嘧啶^[40]^	14 d为1个周期，共4个周期
	SC/VB6+卡培他滨	SC/VB6 20-40 mL+0.9%氯化钠注射液250 mL，静脉滴注，*qd*，14 d^[41]^	21 d为1个周期，共3个周期
乳腺癌			
联合化疗	SC/VB6+TEC方案	SC/VB6 50 mL+0.9%氯化钠注射液或5%葡萄糖注射液250 mL，静脉滴注，*qd*， 14 d+多西他赛+表柔比星+环磷酰胺^[42-44]^	21 d为1个周期，共2个周期
术后辅助，联合放疗	SC/VB6+放疗	SC/VB6 30 mL+0.9%氯化钠注射液，静脉滴注，*qd*，15 d+放疗^[45]^	15 d，与放疗同步
卵巢癌			
联合化疗	SC/VB6+TP方案	SC/VB6 50 mL+0.9%氯化钠注射液或5%葡萄糖注射液250 mL，静脉滴注，*qd* +紫杉醇+顺铂^[46]^	21 d为1个周期，共6-8个周期

SC/VB6规格10 mL：0.1 mg。SC/VB6：斑蝥酸钠维生素B6注射液；*qd*：每日1次；bid：每日2次；TACE：经导管动脉化疗栓塞术；MVA：微波消融；GP：吉西他滨+顺铂；GC：吉西他滨+卡铂；TP：紫杉醇+顺铂；NP：长春瑞滨+顺铂；DP：多西他赛+顺铂；DC：多西他赛+卡铂；AP：培美曲塞+顺铂；FOLFOX4：奥沙利铂+亚叶酸钙+5-氟尿嘧啶；TEC：多西他赛+表柔比星+环磷酰胺。

### 3.2 肺癌中的临床应用

推荐意见4：对于接受系统化疗的非小细胞肺癌（non-small cell lung cancer, NSCLC）患者，推荐联合斑蝥酸钠维生素B6注射液（IB）。对于合并恶性胸腔积液（malignant pleural effusion, MPE）的NSCLC患者，推荐采用化疗联合斑蝥酸钠维生素B6注射液进行胸腔灌注，以增强局部控制（IB）。

晚期肺癌的治疗强调以全身性治疗为核心的多学科综合模式。近年来，在分子靶向治疗与免疫治疗的推动下，其治疗策略持续演进，患者生存结局得到明显改善。尽管如此，对于驱动基因阴性或处于特定治疗线数的患者而言，传统含铂双药化疗仍构成重要的治疗基础。在此背景下，斑蝥酸钠维生素B6注射液在联合治疗方案中展现出增强抗肿瘤疗效的作用，同时减轻放化疗相关的毒性反应，优化晚期NSCLC的治疗效果。

#### 3.2.1 联合化疗

含铂双药化疗是我国晚期NSCLC的常用治疗方案，但常伴随一定的不良反应。多项研究讨论了在吉西他滨/铂类（GP方案）^[7,9,10]^、紫杉醇/铂类（TP方案）^[13,14]^、长春瑞滨/铂类（NP方案）^[15-17]^、多西他赛/铂类（DP方案）^[18,19]^、培美曲塞/铂类（AP方案）^[20-22]^等标准含铂双药方案基础上，联合应用斑蝥酸钠维生素B6注射液的效果。系列研究结果显示，联合斑蝥酸钠维生素B6注射液在缓解骨髓抑制、消化道反应等化疗相关毒性方面具有一定优势，并显示出改善患者生活质量和临床症状的趋势。

一项纳入19项随机对照试验的*meta*分析提供了较为综合的证据^[4]^，结果显示，与单纯化疗相比，化疗联合斑蝥酸钠维生素B6注射液在客观缓解率（objective response rate, ORR）（RR=1.58, 95%CI: 1.40-1.79）、卡氏体能状态（Karnofsky performance status, KPS）评分改善（RR=1.68, 95%CI: 1.42-1.99）、临床症状改善（RR=1.68, 95%CI: 1.44-1.96）等方面表现更优，同时降低了白细胞减少（RR=0.36, 95%CI: 0.27-0.49）、血小板较少（RR=0.46, 95%CI: 0.33-0.63）及呕吐（RR=0.50, 95%CI: 0.37-0.67）的发生风险。Cando-L1研究是一项开放标签、随机III期临床研究^[47]^，其初步报告显示，在表皮生长因子受体（epidermal growth factor receptor, *EGFR*）野生型或未知晚期/转移性NSCLC患者的二线治疗中，斑蝥酸钠维生素B6注射液联合多西他赛组患者的中位总生存期（overall survival, OS）显著优于多西他赛单药组（9.83 *vs* 5.03个月，*P*=0.035）。

#### 3.2.2 胸腔灌注

MPE是晚期恶性肿瘤的常见并发症，系肿瘤细胞侵犯胸膜所致的胸膜腔积液。MPE几乎可发生于所有恶性肿瘤，其中肺癌是导致MPE最常见的病因^[48]^。《肺癌合并恶性胸腔积液诊疗专家共识》^[48]^建议将化疗药物或联合其他药物进行胸腔灌注治疗作为MPE管理的重要手段。

多项临床研究评估了斑蝥酸钠维生素B6注射液用于MPE局部灌注治疗的效果。研究^[25,26]^显示，斑蝥酸钠维生素B6注射液联合顺铂行胸腔灌注，可显著提高肺癌合并MPE患者的积液缓解率，改善生活质量。斑蝥酸钠维生素B6注射液联合洛铂胸腔灌注也观察到同样的获益^[27]^。

因此，斑蝥酸钠维生素B6注射液单药^[49]^或联合化疗药物^[26,27,50]^胸腔灌注对控制MPE更加明显、迅速，临床效果显著，可改善患者体能状态，胃肠道反应发生率更低，提升患者免疫力^[50]^，提高患者生活质量。

#### 3.2.3 其他联合治疗

免疫检查点抑制剂（immune checkpoint inhibitors, ICIs）的应用为晚期驱动基因阴性NSCLC的治疗带来重大变革。斑蝥酸钠维生素B6注射液作为一种具有免疫调节作用的药物，可增强机体的抗肿瘤免疫应答。研究^[51,52]^表明，该药物与程序性细胞死亡受体1（programmed cell death 1, PD-1）抗体联合化疗方案，可降低肿瘤标志物水平，改善血清炎症因子谱，并有助于提升患者生活质量及体能状态，整体安全性良好。

放疗在晚期NSCLC中主要用于局部病灶控制与症状缓解，并可延缓耐药发生、降低远处转移风险。临床研究^[24]^表明，放疗期间联合应用斑蝥酸钠维生素B6注射液，有助于降低放射性肺炎及骨髓抑制的发生风险，并减轻放疗相关毒副反应。

目前，斑蝥酸钠维生素B6注射液在联合免疫治疗及放疗领域的研究证据仍相对有限，且多为观察性研究。因此，其在减轻放射性损伤、增强免疫治疗效果方面的具体作用机制及最佳应用模式，尚待更多大样本、前瞻性研究进一步验证。

### 3.3 原发性肝细胞癌（hepatocellular carcinoma, HCC）中的临床应用

推荐意见5：对于接受系统化疗的原发性HCC患者，推荐联合使用斑蝥酸钠维生素B6注射液（IB）。经导管动脉化疗栓塞术（transcatheter arterial chemoembolization, TACE）中，建议联合斑蝥酸钠维生素B6注射液（IB）。接受放疗的原发性HCC患者，可在放疗期间联合斑蝥酸钠维生素B6注射液（IIC）。晚期原发性HCC患者可以考虑接受斑蝥酸钠维生素B6注射液单药治疗（IIB）。

HCC的治疗策略强调多学科协作，综合运用手术、消融、介入、放疗及系统性抗肿瘤治疗等多种手段。体外研究^[53]^证实在4种HCC细胞系中，斑蝥酸钠维生素B6注射液可阻滞多种人HCC细胞周期进程，并促进细胞凋亡。因此，斑蝥酸钠维生素B6注射液与上述治疗方式联用，可增强抗肿瘤治疗效果，减轻其他治疗相关毒性，改善患者生活质量。

#### 3.3.1 联合全身化疗

FOLFOX4方案在我国被批准用于不适合手术切除或局部治疗的局部晚期和转移性HCC的一线治疗方案^[54]^。一项斑蝥酸钠维生素B6注射液同步低剂量放疗序贯FOLFOX4方案治疗晚期HCC^[34]^，结果显示，患者中位OS为9.7个月，中位无进展生存期（progression-free survival, PFS）为5.9个月，其中单发巨块型患者的中位OS明显优于肝内多发转移患者，提示该联合方案能提高单纯FOLFOX4方案的疗效，且不良反应可控。蒋峰等^[35]^的研究进一步证实，在5-氟尿嘧啶+奥沙利铂方案基础上联合斑蝥酸钠维生素B6注射液，可显著延长患者生存时间。该研究将64例患者随机分组，结果显示，治疗组的24个月生存率达到50%，显著高于对照组的6.67%（*P*<0.05），提示联合用药有助于提高近期疗效并延长生存时间。同时，治疗组患者在生活质量和体能状态的改善方面也优于对照组。

此外，斑蝥酸钠维生素B6注射液联合化疗还可改善HCC患者的免疫功能，具体表现为CD3^+^、CD4^+^ T细胞比例及CD4^+^/CD8^+^比值升高，并有助于改善患者临床症状、增加体重，效果均优于单纯化疗^[30]^。一项*meta*分析^[55]^结果显示，与单纯化疗或对症支持治疗相比，联合斑蝥酸钠维生素B6注射液可提高HCC患者的近期疗效与生活质量，并减轻化疗相关的胃肠道反应、骨髓抑制及肝功能损伤等不良反应。

#### 3.3.2 联合TACE治疗

TACE是不可手术切除HCC最常用的局部治疗方法之一，具有微创、可重复等优点^[54]^。然而，TACE术后常出现恶心、呕吐、白细胞减少及肝功能损伤等不良反应^[32]^。研究显示，TACE术前^[30]^或术后^[31,32]^静脉滴注斑蝥酸钠维生素B6注射液，可以显著减轻患者恶心、呕吐等胃肠道反应。此外，在TACE术中，与化疗药物序贯使用斑蝥酸钠维生素B6注射液50-80 mL^[28,29]^，有助于减少化疗药物的毒副反应，并显示出延长生存时间、提高远期生存率的趋势。

索拉非尼是多靶点抗肿瘤药物，TACE联合索拉非尼是目前临床治疗HCC的常用策略。徐雪勤等^[33]^在此方案基础上加用斑蝥酸钠维生素B6注射液，结果显示，观察组患者血清肿瘤标志物高尔基体跨膜糖蛋白73（Golgi protein 73, GP73）、甲胎蛋白（alpha-fetoprotein, AFP）、糖类抗原19-9（carbohydrate antigen 19-9, CA19-9）下降幅度更为显著，肝功能指标血清总胆红素（total bilirubin, TBiL）、谷丙转氨酶（alanine aminotransferase, ALT）和白蛋白（albumin, ALB）改善更优，安全性方面两组总体耐受性良好，表明三药联合可更有效抑制肿瘤进展、改善肝功能，且安全性良好。

一项*meta*分析^[56]^纳入7项随机对照试验共562例患者，分析结果进一步证实，斑蝥酸钠注射液联合TACE治疗HCC，相较单纯TACE，在提高治疗有效率、临床受益率及生存率方面均更具有优势。

#### 3.3.3 联合放疗

斑蝥酸钠维生素B6注射液与放疗联用可发挥协调增效与减毒效应^[36,57]^。薄常文等^[57]^的研究显示，与单纯放疗相比，联合斑蝥酸钠维生素B6注射液可显著提高原发性HCC患者的ORR（80% *vs* 55.3%, *P*<0.05），并降低骨髓抑制发生率，表明联合方案在促进肿瘤消退、提高近期疗效的同时，还能有效保护骨髓功能。

刘建民等^[36]^针对中晚期HCC患者的研究进一步证实，斑蝥酸钠维生素B6注射液联合射波刀治疗，在近期疗效与生活质量改善方面均优于单纯射波刀治疗，且急性不良反应发生率更低，提示该联合方案有助于病情稳定、延缓疾病进展。此外，斑蝥酸钠维生素B6注射液联合微波消融术也显示出协同增效与减轻不良反应的作用^[37]^。

#### 3.3.4 单药治疗

在单药应用方面，一项前瞻性单中心III期临床研究^[38]^证实，斑蝥酸钠维生素B6注射液可有效改善晚期HCC患者的临床症状与生活质量，并延长生存时间，同时展示出良好的安全性。

### 3.4 其他癌种的探索性应用

推荐意见6：对于常规治疗方案受限或疾病进展的晚期实体瘤及血液系统恶性肿瘤患者，可考虑参加斑蝥酸钠维生素B6注射液相关临床研究（IIC）。

斑蝥酸钠维生素B6注射液已用作多种消化系统肿瘤的抗肿瘤药物，例如胃癌^[41,58,59]^、结直肠癌^[39-40,60]^和贲门癌^[61]^。斑蝥酸钠维生素B6注射液联合XELOX方案给胃癌患者带来更好的治疗获益，同时减轻XELOX方案相关的白细胞减少、恶心和呕吐等不良反应^[62]^。同样，斑蝥酸钠维生素B6注射液联合FOLFOX方案治疗胃癌，能有效缓解单用FOLFOX方案引起的胃肠道反应和肝功能损伤^[63]^。一项对24项试验中1825例晚期消化系统肿瘤患者进行的荟萃分析^[64]^表明，与单纯常规药物治疗相比，联合斑蝥酸钠维生素B6注射液治疗在提高总体肿瘤缓解率、疾病控制率和体能状态方面更有效，同时能减轻化疗引起的不良反应。

对于非消化系统肿瘤，斑蝥酸钠维生素B6注射液在临床上常与化疗联用于乳腺癌^[42-44]^和卵巢癌^[46]^，或与放化疗联用于宫颈癌^[65,66]^和鼻咽癌^[67,68]^。临床前研究^[69]^表明，斑蝥酸钠能有效抑制乳腺癌细胞增殖、侵袭和迁移，通过磷脂酰肌醇3-激酶（phosphatidylinositol 3-kinase, PI3K）/蛋白激酶B（protein kinase B, AKt）/哺乳动物雷帕霉素靶蛋白（mammalian target of rapamycin, mTOR）通路诱导G_0_/G_1_期细胞周期停滞和凋亡。斑蝥酸钠不仅能抑制宫颈癌细胞生长，还能通过抑制蛋白酪氨酸磷酸酶非受体型1（protein tyrosine phosphatase non-receptor type 1, PTPN1）增强其对顺铂的敏感性^[70]^。斑蝥酸钠维生素B6注射液在临床上还与化疗联合用于治疗血液系统恶性肿瘤^[71,72]^。

目前对于斑蝥酸钠维生素B6注射液在这些癌种中的获益-风险特征，仍然缺乏全面的了解，有必要进行系统评价和荟萃分析，并为斑蝥酸钠维生素B6注射液在肿瘤治疗中的合理临床应用提供循证支持。

### 3.5 化疗所致白细胞减少症

推荐意见7：建议化疗时联合应用斑蝥酸钠维生素B6注射液，可改善化疗导致的骨髓抑制，降低白细胞减少发生率（IB）。

肿瘤化疗导致的白细胞减少是化疗常见的血液学不良反应和剂量限制性不良反应。白细胞降低的程度与使用化疗药物的种类和剂量有关。当使用5-氟尿嘧啶、吉西他滨、紫杉类等细胞周期特异性药物时，中性粒细胞计数的最低点通常见于给药后7-14 d，并于14-21 d逐渐恢复至正常值；当使用环磷酰胺、阿霉素等细胞周期非特异性药物时，中性粒细胞计数的最低点通常出现在给药后的10-14 d，于21-24 d逐渐恢复正常^[73]^。

两项*meta*分析^[74,75]^显示化疗联合斑蝥酸钠维生素B6注射液可有效降低白细胞减少发生率，改善患者一般状况。多项临床研究^[14,43,72,76]^结果表明斑蝥酸钠维生素B6注射液与包括细胞周期特异性药物（5-氟尿嘧啶、吉西他滨、紫杉类等）及细胞周期非特异性药物（环磷酰胺、阿霉素等）的化疗方案联合使用都可以有效减轻化疗导致的白细胞减少，提高患者生活质量。

## 4 不良反应及处理建议

推荐意见8：对于轻度的不良反应，如轻微的恶心、呕吐、发热等，可先采取对症治疗。同时，可适当调整药物剂量或用药频率，观察症状是否改善。如果出现中重度不良反应，应立即停药（IB）。

斑蝥酸钠维生素B6注射液常见不良反应包括恶心、呕吐、肌酐/尿素氮升高、发热及静脉炎等，但程度较轻，为中度或者轻度，且均经停药及对症处理后消失。一项真实世界研究纳入的960例患者中，有88例患者出现不良反应，其中26例出现A型恶心、呕吐，9例出现B型肝功能损伤，10例出现B型肾功能损伤，25例出现B型发热，18例出现A型静脉炎，所有患者经停药及对症处理后症状消失。药物引起的恶心呕吐在用药早期出现，一般无需特殊处理。由此可见斑蝥酸钠维生素B6注射液用于癌症治疗的不良反应程度较轻，安全性高^[77]^。

用药前做好患者教育，充分告知患者可能出现的不良反应，使患者对于不良反应有预期性准备。

## 5 总结

斑蝥酸钠维生素B6注射液在多种恶性肿瘤的临床治疗中显示出积极的疗效，在联合治疗中表现出独特优势，能有效减毒增效、改善患者生活质量，丰富了临床用药选择。共识制定本身即为一种推动肿瘤治疗规范化的重要方法论实践，通过系统梳理斑蝥酸钠维生素B6注射液的临床证据与实践经验，为斑蝥酸钠维生素B6注射液在临床的规范化应用提供指导，并期待未来开展更多高质量的临床研究，以进一步优化其在临床治疗中的应用策略。

编写委员会

**主编：**陈军（天津医科大学总医院）潘宏铭（浙江大学邵逸夫医院）邬麟（湖南省肿瘤医院）吴凤英（同济大学附属上海市肺科医院）

**执笔专家（按姓氏汉语拼音排序）：**李昕（天津医科大学总医院）刘京豪（天津医科大学总医院）马晴（天津医科大学总医院）韦森（天津医科大学总医院）张洪兵（天津医科大学总医院）张文学（天津医科大学总医院）

**撰写小组专家（按姓氏汉语拼音排序）：**常香云（天津医科大学总医院）陈恩强（四川大学华西医院）郭克锋（黄河三门峡医院）郭占林（内蒙古医科大学附属医院）黄广慧（山东省立医院）贾军梅（山西医科大学第一医院）宋荣峰（江西省肿瘤医院）王虹（兰州大学第二医院）王克强（天津医科大学总医院）邢培培（天津医科大学总医院）徐燕（北京协和医院）杨立伟（河北医科大学第四医院）姚颐（武汉大学人民医院）邹方文（中南大学湘雅二医院）

## References

[1] Zheng RS, Chen R, Han BF, et al. Cancer incidence and mortality in China, 2022. Zhonghua Zhongliu Zazhi, 2024, 46(3): 221-231. 38468501 10.3760/cma.j.cn112152-20240119-00035

[2] Guyatt GH, Oxman AD, Vist GE, et al. GRADE: an emerging consensus on rating quality of evidence and strength of recommendations. BMJ, 2008, 336(7650): 924-926. doi: 10.1136/bmj.39489.470347.AD 18436948 PMC2335261

[3] Fan MR, Shen Q, Wang DQ, et al. Methodology for clinical practice guidelines-consensus methods for building recommendations. Zhongguo Xunzheng Xinxueguan Yixue Zazhi, 2019, 11(6): 647-653.

[4] Wang Z, Feng F, Wu Q, et al. Disodium cantharidinate and vitamin B 6 injection adjunct with platinum-based chemotherapy for the treatment of advanced non-small-cell lung cancer: a *meta*-analysis. Evid Based Complement Alternat Med, 2019, 2019: 9386273. doi: 10.1155/2019/9386273 30992710 PMC6434307

[5] Peng Y, Chen WZ, Tang L. Review of antitumor drug sodium cantharidate. Guizhou Yike Daxue Xuebao, 2022, 47(10): 1117-1124.

[6] Liu YB, Wei SJ, Yuan SY. Immunomodulatory effects of disodium cantharidinate and vitamin B6 injection against tumors. Shiyong Linchuang Yiyao Zazhi, 2009, 13(23): 56-57.

[7] Li LJ, Liang JW, Zang YR, et al. Curative effect of SCAVB 6 combined with chemotherapy in patients with NSCLC and the impact on quality of life. Aizheng Jinzhan, 2018, 16(11): 1372-1374, 1389.

[8] Wei SJ, Yuan SY, Liu YB. Experimental research of disodium cantharidinate and vitamin B6 injection regulating cytokines *in vitro*. Xiandai Zhongliu Yixue, 2007, 15(9): 1226-1228.

[9] Zeng HX, Guo SJ, Huang P, et al. Clinical observation of disodium cantharidinate and vitamin B 6 injection combined with gemcitabine and carboplatin in the treatment of elderly patients with previously untreated advanced non-small cell lung cancer. Guoji Zhongliuxue Zazhi, 2014, 41(4): 319-320.

[10] Li YQ, Feng GH. Efficacy and quality of life analysis of disodium cantharidinate and vitamin B6 injection in the treatment of advanced non-small cell lung cancer. Zhongguo Linchuang Yanjiu, 2016, 29(12): 1666-1668.

[11] Yan HQ. Analysis of the effectiveness of combined treatment of non-small cell lung cancer with sodium cantharidinate/vitamin B6 injection and chemotherapeutic drugs. Shijie Fuhe Yixue, 2023, 9(11): 16-19.

[12] Wang B, Cui J. Treatment of mid-late stage NSCLC using sodium cantharidinate/vitamin B6/GP regimen in clinic. J Cancer Res Ther 2014, 10 Suppl 1: C79-C81. doi: 10.4103/0973-1482.139771 25207898

[13] Luo Y, Bao ZH, Jiang Y, et al. Clinical observation of sodium cantharidate vitamin B 6 injection combined with chemotherapy in the treatment of lung squamous cell carcinoma. Chongqing Yixue, 2022, 51(19): 3284-3288.

[14] Wang DZ. Efficacy of sodium cantharidin combined with TP themotherapy on advanced non-small cell lung cancer and infLuence on tumor angiogenesis. Yixue Linchuang Yanjiu, 2019, 36(2): 265-267.

[15] Li LJ, Zang YR, Yu WH, et al. Therapeutic effect of chemotherapy combined with sodium cantharidinate vitamin B 6 injection in patients with non-small cell lung cancer and its mechanism. Jilin Daxue Xuebao (Yixueban), 2018, 44(6): 1286-1290.

[16] Zhu XH. Efficacy of disodium cantharidinate combined with chemotherapy in the treatment of advanced non-small cell lung cancer and its effects on immune function and tumor markers. Haerbin Yiyao, 2021, 41(6): 72-73.

[17] Tang FL, Zhang HP. Clincical observation of Aiyishu injection combined with chemotherapy in non small-cell lung cancer. Zhongguo Yiyao Daobao, 2007, 4(26): 46-47.

[18] Jia JM, Li ZH, Li Z. Observation on the efficacy of disodium cantharidinate and vitamin B6 combined with intravenous chemotherapy in the treatment of advanced lung cancer. Guoji Zhongliuxue Zazhi, 2014, 41(1): 77-78.

[19] Sun GL, Ren ZH, Wang DP. Clinical observation of disodium cantharidinate and vitamin B6 injection combined with DC regimen in the treatment of non-small cell lung cancer. Yiyao Qianyan, 2013(32): 145-146.

[20] Chen YL, Yang WB. Effect of disodium cantharidinate and vitamin B6 injection combined with chemotherapy on immune function in patients with non-small cell lung cancer. Zhongguo Laonianxue Zazhi, 2015(20): 5806-5807.

[21] Song Y, Leng CH, Zhou J. Clinical efficacy of disodium cantharidinate and vitamin B6 injection as an adjuvant to chemotherapeutic drugs in the treatment of lung cancer. Linchuang Heli Yongyao Zazhi, 2022, 15(21): 73-76.

[22] Yao J, Yang ZH, Zhao XJ, et al. Clinical observation of sodium cantharidate vitamin B 6 combined with chemotherapy on advanced non-small cell lung cancer. Hebei Zhongyi, 2018, 40(7): 1005-1009, 1039.

[23] Luo Y, Wang HZ, Wang JY, et al. Clinical observation of rh-endostatin combined with cantharidin sodium vitamin B 6 in the treatment of advanced non-small cell lung cancer. Zhongguo Yaofang, 2016, 27(26): 3668-3670, 3671.

[24] Wang LZ, Zhang HJ, Guo Y, et al. Clinical evaluation of cantharidate sodium vitamin B 6 injection combined with three-dimensional conformal radiotherapy in treatment of advanced non-small cell lung cancer in the elderly. Dayisheng, 2019, 4(2): 3-4, 7.

[25] Zhou P, Wu H, Lu B. Efficacy and safety of disdium cantharidinate and vitamin B6 combined with chemotherapy drugs intrapleural thermal perfusion in the treatment of lung cancer complicated with malignant pleural effusion. Linchuang Heli Yongyao, 2024, 17(3): 22-25.

[26] Wu ZG. Therapeutic effects of sodium cantharidate vitamin B6 injection combined with cisplatin on patients with lung cancer complicated with pleural effusion. Zhongguo Shiyong Yikan, 2019, 46(5): 119-121.

[27] Wang ZW, Hu EB, Li L. Comparison of the efficacy of lobaplatin combined with disodium cantharidinate and vitamin B6 injection by thoracic perfusion versus intravenous systemic chemotherapy in the treatment of malignant pleural effusion. Shijie Zhongyiyao, 2017, 12(S2): 151-152.

[28] Tian XL, Yang P. Clinical efficacy of treatment of liver cancer with combined sodium cantharidate vitamin B6 injection and TACE therapy. Zhongguo Zhongliu Linchuang Yu Kangfu, 2006, 13(4): 351-353.

[29] Wei YF, Wu JS, Huo ZG, et al. Evaluation of the efficacy of disodium cantharidinate and vitamin B 6 injection combined with interventional embolization chemotherapy in the treatment of 48 cases of intermediate and advanced primary liver cancer. Zhongguo Yaoye, 2015(5): 72-74.

[30] Zhang MJ, Zuo CF. Clinical efficacy of Sodium Cantharidate Vitamin B6 Injection combined with chemotherapy in treatment of liver cancer. Shiyong Zhongliu Zazhi, 2011, 26(1): 50-52.

[31] Zhang B. Clinical observation of Aiyishu injection combined with TACE in the treatment of hepatocellular liver cancer. Zhongguo Yiyao Daobao, 2007, 4(26): 48-49.

[32] Yang F, Yang J, Pu ZJ, et al. Venous administration of vitamin B 6 sodium cantharidate after TACE in the treatment of patients with primary liver cancer. Shiyong Ganzangbing Zazhi, 2024, 27(2): 263-266.

[33] Xu XQ, Zhao YX, Wang JF. Short-term efficacy and safety of sorafenib combined with disodium cantharidinate vitamin B6 in patients with transcatheter arterial chemoembolization for primary liver cancer. Zhongguo Heli Yongyao Tansuo, 2020, 17(8): 82-87.

[34] Xu YC, Chen KQ, Liang WC, et al. Clinical observation of disodium cantharidinate and vitamin B 6 injection combined with low-dose radiother-apy sequenced by FOLFOX4 regimen for advanced hepatocellular carcinoma. Linchuang Zhongliuxue Zazhi, 2020, 25(1): 33-39.

[35] Jiang F, Sun XH, Gao YW, et al. Effects of sodium cantharidate vitamin B 6 injection combined with different chemo-therapeutic drugs on rbm38, spats2 and prognosis in patients with advanced liver cancer. Zhongxiyi Jiehe Ganbing Zazhi, 2023, 33(3): 237-241.

[36] Liu JM. Clinical observation of middle-late stage primary hepatic carcinoma patients treated by disodium cantharidinate and vitamin B6 injection combined Cyberknife therapy. Xiandai Zhongliu Yixue, 2015, 23(3): 392-394.

[37] Liang D, Hu J, Wang QC. Observation on the efficacy of microwave ablation combined with disodium cantharidinate and vitamin B6 injection in the treatment of primary liver cancer. Ganzang, 2019, 24(10): 1205-1207.

[38] Liang Y, Liang WC, Feng J, et al. A prospective single-center phase III clinical study of disodium cantharidinate and vitamin B 6 in the treatment of advanced primary liver cancer. Zhongguo Zhongliu Linchuang Yu Kangfu, 2014, 21(2): 135-138.

[39] Shi XY, Zhang J, Meng W, et al. Clinical observation of sodium cantharidinate and vitamin B 6 injection combined with XELOX protocol in treatment of patients with advanced colorectal carcinoma. Jiefangjun Yiyao Zazhi, 2017, 29(6): 36-39.

[40] Chen Y, Zhu WM, Zhou DX, et al. Clinical study on disodium cantharidinate and vitamin B 6 combined with raltitrexed and oxaliplatin in treatment of advanced colorectal cancer. Xiandai Yaowu Yu Linchuang, 2016, 31(11): 1784-1787.

[41] Wang YW, Yang RL. The effect observation of sodium cantharidate vitamin B6 combined with capecitabine in the treatment of advanced stomach and colorectal carcinoma. Zhongguo Yixue Chuangxin, 2017, 14(36): 23-26.

[42] Quan CY, Qiu XP, Zhang ZQ, et al. Efficacy of disodium cantharidinate and vitamin B 6 in the treatment of advanced breast cancer and its impact on quality of life and safety. Zhongguo Putong Waike Zazhi, 2019, 28(5): 636-640.

[43] Liang JW, Li LJ, Yu WH, et al. The real world study on the effect of SCAVB 6 combined with chemotherapy on the short-term efficacy and adverse reactions of breast cancer. Hebei Yixue, 2019, 25(4): 580-582.

[44] Zhou R, Dong HM, Huang JK, et al. Effect of cantharis acid sodium vitamin B 6 injection on youth postoperative chemotherapy patients with breast cancer. Anhui Yiyao, 2018, 22(8): 1575-1578.

[45] Xing ZY, Zhang JB, Wu RF, et al. Observation on the short-term efficacy of disodium cantharidinate combined with three-dimensional conformal radiotherapy in the treatment of breast cancer. Guoji Zhongliuxue Zazhi, 2015, 42(1): 77-78.

[46] Li Y, Zhang T, Guo YP, et al. Effects of cantharidin sodium vitamin B 6 combing with PT therapy on the lymphatic growth factor of ovarian cancer tissues. Hainan Yixueyuan Xuebao, 2015, 21(12): 1701-1703, 1706.

[47] Wu L, Deng C, Zhang H, et al. Efficacy and safety of docetaxel and sodium cantharidinate combination *vs.* either agent alone as second-line treatment for advanced/metastatic NSCLC with wild-type or unknown EGFR status: An open-label, randomized controlled, prospective, multi-center phase III trial (Cando-L1). Front Oncol, 2021, 11: 769037. doi: 10.3389/fonc.2021.769037 34976813 PMC8715707

[48] China Association of Health Promotion and Education, Cancer Rehabilitation and Palliative Treatment Professional Committee of China Anti-Cancer Association. Expert consensus on diagnosis and treatment of malignant pleural effusion caused by lung cancer. Zhonghua Zhongliu Zazhi, 2024, 46(1): 40-47. 38246779 10.3760/cma.j.cn112152-20231130-00344

[49] Liu YL, Yang ZF, Zeng L. Therapeutic effect of disodium cantharidinate and vitamin B6 injection on malignant pleural effusion and ascites. Zhonghua Zhongliu Fangzhi Zazhi, 2006, 13(19): 1519-1520.

[50] Xiong X, Shi F, Yang YM, et al. Observation on the effect of disdium cantharidinate and vitamin B 6 injection combined with cisplatin in the treatment of malignant pleural effusion. Zhongguo Yixue Chuangxin, 2024, 21(1): 35-39.

[51] Lv YD, Yang YH, Lv BL. Analysis of the effect of pembrolizumab combined with disodium cantharidinate and vitamin B6 injection in the treatment of patients with lung squamous cell carcinoma. Yixue Lilun Yu Shijian, 2024, 37(12): 2038-2040.

[52] Zhao ZC, Xie ZX, Su Y, et al. Effect observation of camrelizumab combined with disodium cantharidinate and vitamin B 6 in the treatment of patients with non-small cell lung cancer. Yishi Zaixian, 2024, 14(2): 42-44.

[53] Si LL, Xu W, Li L, et al. Effect of sodium cantharidinate and vitamin B 6 injection on human hepatocellular carcinoma cells and its mechanism. Jiefangjun Yixue Zazhi, 2025, 50(6): 747-755.

[54] Department of Medical Administration of National Health Commission. Guidelines for diagnosis and treatment of primary liver cancer (2024). Xiehe Yixue Zazhi, 2024, 15(3): 532-558.

[55] Wang XF, Luo XH. Adjuvant treatment with disodium cantharidinate and vitamin B6 injection for patients with hepatocellular carcinoma: a *meta* analysis based on randomized controlled trials. Guoji Zhongliuxue Zazhi, 2014, 41(1): 69-73.

[56] Xu Z, Cao H, Bai BJ. Meta-analysis of disodium cantharidinate injection combined with transcatheter arterial chemoembolization in the treatmemt of hepatocellular carcinoma. Zhongguo Shenghua Yaowu Zazhi, 2015(5): 66-71.

[57] Bo CW, Li N, An YH, et al. Comparative analysis of the clinical efficacy of intensity-modulated radiotherapy alone versus combined with disodium cantharidinate and vitamin B 6 injection in the treatment of primary liver cancer. Guoji Zhongliuxue Zazhi, 2014, 41(1): 79-80.

[58] Gan YH, Li YJ, Li FX, et al. Effects of disodium cantharidinate and vitamin B 6 injection combined with SOX regimen on miR-18a, miR-34a and immune cell counts in patients with advanced gastric cancer. Xiandai Xiaohua Ji Jieru Zhenliao, 2021, 26(12): 1551-1555.

[59] Xie ZX, Wang JS, Liu YB. Observation of the short-term efficacy and adverse reactions of disodium cantharidinate and vitamin B6 injection combined with chemotherapy in the treatment of advanced gastric cancer. Hebei Yiyao, 2016, 38(10): 1554-1556, 1559.

[60] Liu T, Liu D, Liu XC, et al. *Meta* analysis of evaluating the effectiveness and safety of disodium cantharidinate and vitamin B6 injection plus chemotherapy compared with chemotherapy alone in the treatment of colorectal cancer. Zhongguo Yikan, 2018, 53(10): 1114-1117.

[61] Zhu H, Zhang HY, He M. Effect of disodium cantharidinate and vitamin B6 injection combined with chemotherapy on plasma D-dimer l Level in patients with advanced cardia cancer. Zhongliu Fangzhi Yanjiu, 2015, 42(2): 168-170.

[62] Zhang D, Wu J, Wang K, et al. Which are the best Chinese herbal injections combined with XELOX regimen for gastric cancer?: A PRISMA-compliant network *meta*-analysis. Medicine (Baltimore), 2018, 97(12): e0127. doi: 10.1097/MD.0000000000010127 PMC5895335 29561411

[63] Zhang D, Zheng J, Ni M, et al. Comparative efficacy and safety of Chinese herbal injections combined with the FOLFOX regimen for treating gastric cancer in China: a network *meta*-analysis. Oncotarget, 2017, 8(40): 68873-68889. doi: 10.18632/oncotarget.20320 28978164 PMC5620304

[64] Liu M, Xu C, Sun Y. Efficacy and safety of sodium cantharidinate and vitamin B6 injection for the treatment of digestive system neoplasms: a *meta*-analysis of randomized controlled trials. Drug Des Devel Ther, 2018, 13: 183-203. doi: 10.2147/DDDT.S190674 PMC6312696 30643386

[65] Jiang HL, Huang H. Effect of sodium cantharidate vitamin B6 Injections in adjuvant treatment of advanced cervical cancer. Zhongguo Yaoshi, 2014(11): 1904-1905, 1906.

[66] Jin H, Zhang YY, Li Q. Therapeutic effect of concurrent chemoradiotherapy combined with sodium cantharidate vitamin B6 injection for advanced cervical cancer. Shiyong Linchuang Yiyao Zazhi, 2013, 17(19): 149-151.

[67] Mi ZT, Zang ZF, Zhang XF, et al. Effect observation of sodium cantharidate and vitamin B 6 combined with concurrent chemoradiotherapy in treatment of locally advanced nasopharyngeal carcinoma. Zhongliu Yanjiu Yu Linchuang, 2017, 29(4): 262-265.

[68] Ying XZ, Xu YP, Wu H, et al. Clinical application value of disodium cantharidinate and vitamin B 6 chemotherapy in nasopharyngeal carcinoma. Zhongguo Shenghua Yaowu Zazhi, 2016(7): 141-144.

[69] Pang JL, Xu LS, Zhao Q, et al. Sodium cantharidate promotes autophagy in breast cancer cells by inhibiting the PI3K-Akt-mTOR signaling pathway. Front Pharmacol, 2022, 13: 1000377. doi: 10.3389/fphar.2022.1000377 36408240 PMC9666387

[70] Chen X, Zhou M, Fan W, et al. Combination of sodium cantharidinate with cisplatin synergistically hampers growth of cervical cancer. Drug Des Devel Ther, 2021, 15: 171-183. doi: 10.2147/DDDT.S282777 PMC7812528 33469269

[71] Zhong M, Xu QL, Su QH. Effect of cantharis acid sodium vitamin B6 combined with CHOP chemotherapy on non-Hodgkin's lymphoma. Hainan Yixueyuan Xuebao, 2014, 20(6): 782-785.

[72] Wu XY, Lin WY, Nong QW, et al. Clinical observation of sodium cantharidate vitamin B 6 injection combined with chemotherapy for acute leukemia patients. Guoji Zhongliuxue Zazhi, 2014, 41(1): 74-76.

[73] China Anti-Cancer Association Tumor Clinical Chemotherapy Professional Committee, China Anti-Cancer Association Tumor Support Therapy Professional Committee. Consensus on clinical diagnosis, treatment, and prevention of chemotherapy-induced neutropenia in China (2023). Zhonghua Zhongliu Zazhi, 2023, 45(7): 575-583. 37460439 10.3760/cma.j.cn112152-20230224-00076

[74] Dai LL. *Meta*-analysis of the clinical efficacy of disodium cantharidinate and vitamin B6 combined with chemotherapy in the treatment of NSCLC. Shiyong Zhongliu Zazhi, 2012, 27(6): 643-645.

[75] Sheng L, Li Y, Chen JP. Disodium cantharidinate and vitamin B6 injection plus chemotherapy for non-small cell lung cancer: A systematic review. Zhongguo Xunzheng Yixue Zazhi, 2012, 12(5): 589-595.

[76] Jian RN, Zhao XM, Lv ZR, et al. *Meta*-analysis of clinical efficacy and safety of disodium cantharidinate combined with conventional chemotherapy in the treatment of non-small cell lung carcinoma. Zhongguo Heli Yongyao Tansuo, 2024, 21(10): 43-51.

[77] Li LJ, Zang YR, Liang JW, et al. Real world study on efficacy and safety of cantharidin and vitamin B6 injection. Zhongliu Yaoxue, 2018, 8(5): 799-802, 807.

